# Cryo-EM as a powerful tool for drug discovery: recent structural based studies of SARS-CoV-2

**DOI:** 10.1186/s42649-021-00062-x

**Published:** 2021-09-25

**Authors:** Han-ul Kim, Hyun Suk Jung

**Affiliations:** Department of Biochemistry, College of Natural Sciences, Kangwon National University, 1, Kangwondaehak-gil, Chuncheon-si, 24341 Gangwon-do Korea

**Keywords:** Transmission electron microscopy, Cryo-electron microscopy, Structure-based drug design, Fragment-based drug discovery, Severe acute respiratory syndrome coronavirus 2

## Abstract

The novel coronavirus, severe acute respiratory syndrome coronavirus 2 (SARS-CoV-2) has arisen as a global pandemic affecting the respiratory system showing acute respiratory distress syndrome (ARDS). However, there is no targeted therapeutic agent yet and due to the growing cases of infections and the rising death tolls, discovery of the possible drug is the need of the hour. In general, the study for discovering therapeutic agent for SARS-CoV-2 is largely focused on large-scale screening with fragment-based drug discovery (FBDD). With the recent advancement in cryo-electron microscopy (Cryo-EM), it has become one of the widely used tools in structural biology. It is effective in investigating the structure of numerous proteins in high-resolution and also had an intense influence on drug discovery, determining the binding reaction and regulation of known drugs as well as leading the design and development of new drug candidates. Here, we review the application of cryo-EM in a structure-based drug design (SBDD) and in silico screening of the recently acquired FBDD in SARS-CoV-2. Such insights will help deliver better understanding in the procurement of the effective remedial solution for this pandemic.

## Introduction

In biological environments, proteins do not statically function, interacting with various binding partners in the context of complex networks and have dynamic regulatory mechanisms. Simultaneously, individual proteins perform with several partners and change the assembly state of the complex (Berendsen and Hayward 2000). Namely, the structural study is required for a comprehensive understanding of the complex function of high-molecular proteins (Dubrovsky et al. 2015). The recent revolution in cryo-electron microscopy (cryo-EM) has provided an outburst of structure determinations at better high-resolution (Kuhlbrandt 2014). This has allowed cryo-EM results to yield visualization of native molecular interactions such as ligand binding state, molecular complex and so on, and these results have provided unparalleled insights into the molecular mechanisms of physiological processes (Scapin et al. 2018). An adoption of structural biology in small molecule related drug discovery is well established in now. The consequence of structural study in drug discovery has been understood since 1980s, and, by the early 1990s the first successful cases were identified (Dorsey et al. 1994; Erickson et al. 1990; Roberts et al. 1990). Over the past two decades, X-ray crystallography has advanced to the magnitude the crystal structures of proteins or small complexes of protein–ligand could be generally resolved with near-atomic resolution (~ 3.0 Å) to contribute screening of drug candidate of small fragment and fragment-based drug discovery (FBDD) (Maveyraud and Mourey 2020). However, this technique requires protein crystallization step from enormous concentration of protein samples, which means there is certain limitation in challenging targets such as membrane proteins and molecular complexes since it is hard to purify and crystallize (Acharya and Lloyd 2005; Li and Sun 2017). As mentioned above, a revolution in cryo-EM has occurred in the past few years, owing to dramatic access in the technological improvement of the hardware and software (Egelman 2016). Thus, cryo-EM has risen as a complementary method to traditional techniques and furnished several advantages. For example, an achievement to larger molecular complex that have been impossible to determinate by X-ray crystallography or nuclear magnetic resonance (NMR) (Frank 2017). Therefore, this improved structural determination of the protein and molecular complex by cryo-EM with near atomic-resolution structure aiding to promote drug development and support structure-based drug design (SBDD) in pharmaceutical industries now and in the near future. The global world is currently suffering from severe economic and health devastation from severe acute reparatory syndrome 2 (SARS-CoV-2) outbreak in Wuhan, China (Lai et al. 2020; Pan et al. 2020a). Vaccine development for this pandemic has been completed by enthusiastic researchers from various pharmaceutical companies such as Pfizer, AstraZeneca, Moderna and so on (Chagla 2021; Knoll and Wonodi 2021; Mahase 2020). Moreover, global vaccinations are underway to prevent infection of it, but the development of certain treatments for remedy is still ongoing. Further, scientists around the world are putting much effort into finding a therapeutic agent (Asai et al. 2020; Petushkova and Zamyatnin 2020; Zafar et al. 2020). This is achieved by analyzing whether it can be applied to SARS-CoV-2 by referring to previously published small molecules, such as by inhibiting replication of same type of viruses and comparing efficacy by screening similar small molecules on the large-scale screening methods (Caly et al. 2020; Jiang et al. 2021; Zhang et al. 2020b). In particular, FBDD is extensively applying on analysis of binding capabilities using molecular dynamics (MD) at in silico environment for selected small molecule candidates after screening the efficacy of the substantive large-scale screening with a preliminary candidate group from database (Chen et al. 2021; Muhseen et al. 2020; Sepay et al. 2021). In this review, we will focus on recent studies of the structural determination of SARS-CoV-2 by cryo-EM and discuss SBDD based on FBDD at in silico screening environment for the development of therapeutic agents for SARSC-CoV-2. We hope that this global pandemic will end soon with various approaches of the numerous researchers in drug development.

## Cryo-EM in drug discovery

The exponential advancement in electron microscopy (EM) have allowed an increase in resolution for efficient drug discovery (Merk et al. 2016). While all of EM analysis has been in use for approximately 50 years to observe biological specimens, only in the past a few years has the result accomplished defining high-resolution structures useful for application in the field of drug discovery (Renaud et al. 2018). Jacques Dubochet, Joachim Frank and Richard Henderson was awarded the Nobel prize in chemistry in 2017, in recognition of their contributions to development of cryo- EM that progress into high-resolution in structural biology using EM. These advancements elucidating the method to promptly capture the native specimen in vitrified ice condition, deconvolute the manifold state of the sample in ice, and increase the performance of the equipment, notably high-sensitive detectors (Nogales 2016). As mentioned above, the improved cryo-EM can provide several advantages than x-ray crystallography, which is typically used in the field of drug discovery with structural studies. First, x-ray crystallography requires crystallized protein samples, a serious bottleneck that is highly restricted in the beginning of many target proteins. On the other hand, cryo-EM is directly observed in native without any modification of the target. Also, it has a wide range of application pool without limitations on macromolecule, virus, and protein complex because large proteins, protein complexes, or flexible samples are difficult to handle in x-ray crystallography. In other words, cryo-EM can observe the native mechanical working state in a target specimen often enabling observation of unexpected drug binding sites or providing an insight to understand the mechanism of molecular regulation. This is robust benefit in SBDD for membrane proteins such as multiple ion channels, transporters and G-protein coupled receptors (GPCR) (Jojoa-Cruz et al. 2018; Pan et al. 2020b; Scheres 2016; Van Drie and Tong 2020; Zhang et al. 2021). Although there are already many papers published for the cryo-EM method, the first step is the preparation of purified samples. This is the same as the starting stage of x-ray crystallography, However the concentration of the samples is usually small as 1 mg/ml (Kwon et al. 2019; Schmidli et al. 2019). Next, optimization procedure is required to obtain the best grid to observe by considering the thickness of the ice, distribution and orientation of the sample and so on. This is a similar step to crystallization of enormous bottle neck in x-ray crystallography (Kampjut et al. 2021). After confirming the quality of grid specimen, the cryo-EM movie images are obtained using the high-performance transmission electron microscope (TEM) and detector. The observed images are refining a three-dimensional reconstruction model employing a typical software according to a particular algorithm in a short time (Bell et al. 2018; Grant et al. 2018; Punjani et al. 2017; Zivanov et al. 2018). The time and cost consumed will be reduced in the future because, the software regarding the image processing step has rapidly improved. Moreover, the results of reconstructed map will also improve (Rohou and Grigorieff 2015; Zhang 2016; Zheng et al. 2017). In addition, these refined cryo-EM models can be used for structural determination fitted with the atomic model of x-ray crystallographic regardless of their resolution, and their own atomic structural modeling is possible at high-resolution (Afonine et al. 2018; Pettersen et al. 2004). Therefore, recent advances in hardware and software have made it easier to demonstrate the results of cryo-EM at ~ 4.0 Å of responsible high-resolution, which is sufficient in identifying the ligand binding state (Zhang et al. 2020a). The highest reconstruction result using cryo-EM is currently 1.2 Å, indicating that cryo-EM has no intrinsic limitations for atomic resolution results of the molecule, it is easy to detect not only the ligand binding but also the small molecule binding at the native water-like environment. suggesting its huge potential as a fascinating tool for SBDD and FBDD (Nakane et al. 2020; Wong et al. 2017; Yip et al. 2020).

## Recent structural studies of SARS-CoV-2 spike proteins using cryo-EM

The coronaviruses are the largest enveloped positive-strand RNA viruses. They have a broad host range in mammalian and avian. It is also the cause to several lesions such as acute and chronic diseases of the respiratory, gastrointestinal, and neurological systems (Wu et al. 2020). The coronavirus spike (S) protein is a highly glycosylated transmembrane fusion protein containing ~ 1400 amino acids as a molecular machine that leads virus particle invade into host cells (Bosch et al. 2003). This S protein involves S1 subunit to bind into the specific target receptor of host cell surface and S2 subunit to conflate into host cell membrane. In addition, S protein can exist in two states, as prefusion and postfusion of structural conformational changes (Li 2016). To invade into the host cells, they have to defeat the surface membrane as a barrier of the host cell through specific target receptor. An individual enveloped virion accomplishes by fusion into membrane surface, mediated by s proteins on their viral membrane (Baker et al. 1999). The molecular and structural study about theses process and protein is required for better understanding to figure out the novel coronavirus in near future. In general, S protein in coronaviruses has been studied as a key target for diagnosis, treatment antibody, and vaccine development. D wrapp et al. determined the prefusion state of SARS-CoV-2 S protein as high-resolution using cryo-EM soon after the begin pandemic (Wrapp et al. 2020). The authors of this paper provided a lot of useful information, as well as high-resolution information about SARS-CoV-2 S protein. First, the authors suggested information to enable structural determination of this mechanical activation by up/down conformation reconstructing the active/inactive state of one monomer’s receptor binding domain (RBD) in intact trimeric structure in the prefusion state of S protein. This has been clearly identified in 3D the classification steps, and the local resolution map of the whole structure suggests that the resolution of this area is extremely lower than others, with significant flexibility (Rawson et al. 2016). Also, the trimer structure that is very similar to the S protein of SARS-CoV in overall shape (Sun et al. 2020). However, the formation of RBD that have a more structurally loose state in closed form. This structural determination supports 10 to 20 times higher affinity for angiotensin-converting enzyme 2(ACE2), the same target receptor as S protein in SARS-CoV. In addition, the authors confirmed the presence of the previously absent sequence RRAR through sequence alignment with SARS-CoV and bat RaTG13. This is a furin recognition sequence region found in critical influenza (Walker et al. 1994; Xia et al. 2020). which the one of suggestive clue that SARS-CoV-2 maybe fatal than before other types. Therefore, this paper can be thought as providing information that is a beginning point for solving this global pandemic. Next, a noticeable recent research paper on SARS-CoV-2 based on structural biology in pharmaceutical field is the stabilization of S protein using specific disulfide and determination of structural study of SARS-CoV-2 S trimer by cryo-EM published by Xiong et al. (2020). In this work, the authors not only suggest effective strategies for structural stability of S trimer, but also identifies the factors known to contribute to structural stability using high-resolution cryo-EM maps. First, the prefusion state of SARS-CoV and Middle East respiratory syndrome (MERS)-CoV are unstable in metastable condition (Kirchdoerfer et al. 2018; Pallesen et al. 2017). Also, SARS-CoV-2 S protein shows similar characterization, and S protein of the SARS-CoV-2 solubilized by detergent can be changed into the postfusion conformation during purification process (Cai et al. 2020; Liu et al. 2020). A suitable immunization to develop successful vaccine need stable antigens, and instability of the prefusion state of SARS-CoV-2 S protein has caused the speed of vaccine development to slow down. In the previous study of stabilization of S protein, researchers inserted SOSIP mutation (a disulfide bond and a stabilizing protein) in the glycoprotein of human immunodeficiency virus-1 (HIV-1) to stabilize the S protein of HIV-1 (Graham et al. 2019). In addition, the authors have tried to SARS-CoV-2 based on their attempts to stabilize structural states by introducing two prolines in the S2 region of former SARS-CoV and MERS-CoV, however have observed that prefusion states cannot be maintained for long term periods (Kirchdoerfer et al. 2018; Pallesen et al. 2017). Thus, their strategy, successful stabilization by engineered disulfide bond adopt into S protein trimer and structural determination by cryo-EM can induce immune response, increase utilization when used as a vaccine, make it available as a diagnostic marker, provide a structural basis for stability for SARS-CoV-2. As a result, this study confirms that the trimer structure of S proteins of SARS-CoV2 maintains structural stability even in long-term storage for 30 days and a high temperature of 60 degrees. This construct showed the best stability compared to the previously known proline mutations or other mutations contributing to structural activity (Xiong et al. 2020). If these results are used for vaccine development or diagnosis reagent, not only will the disadvantages about storage issue of the vaccines currently used will be solved, but their utilization is also expected to be significant, especially in high-temperature areas with poor infrastructure. Currently, the study of SARS-CoV-2 by cryo-EM has also been applied to studies to better understand the overall immune mechanism, focusing on the study of stability and activity of S trimer based on structural information.

## Application of cryo-EM in structure based in silico screening of SARS-CoV-2

‘In silico’ is used to mean experimentation conducted by computer and is related to the other biological environmental terms in vivo and in vitro. This method is generally used to test in vitro data to create an experimental model. Also, such virtual models have employed in the discovery and optimization of small molecules and compound with specific affinity to a biological target, the conformation of absorption, distribution, metabolism, excretion and toxicity properties as well as physicochemical characterization (Ghosh et al. 2006; Kitchen et al. 2004; Leach et al. 2006). Moreover, it means the use of this experimental data in the construct of computational models or virtual simulations can be suggested to make predictions, hypotheses, and to provide achievements in medicine and therapeutics (Rognan 2017). Therefore, this method can be aid to the numerous possible stages of drug discovery in now and future. Currently, rapid and effective development is required using structure based in silico screening without a clear drug development strategy of SARS-CoV-2 being presented. This section explains examples of structure based in silico screening for ligand screening and affinity profiling based on ligand and target, which are currently used mainly in SARS-CoV-2 therapeutic agent discovery/development (Ekins et al. 2007). Recent development of therapeutic agent and discovery of drug candidate for SARS-CoV-2 has led to large-scale screening of previously antiviral-effective compounds or already approved drugs (Caly et al. 2020; Riva et al. 2020; Zhang et al. 2020b). In molecular level, since the structure of S protein of SARS-CoV-2 is already determined by cryo-EM, research has been conducted on molecule levels that exhibit a certain level of affinity to the S protein using existing databases. This appears to be aimed at the more precise regulation by understanding at the molecular level, rather than at the preliminary experimental results, such as by suppressing the replication of virus at the cellular level (Boopathi et al. 2021). These studies are accompanied by the study of in silico environmental molecular dynamics (MD) based on structural biology (Chen et al. 2021; Ibrahim et al. 2020; Sepay et al. 2021). In particular, structure based inhibition of specific main targets are RBD, which causes host cell membrane fusion by contacting the ACE2 receptor in humans, and main protease (M^pro^), which cleave the translated polyprotin from invaded viral RNA and makes it into an individual protein (Erlanson 2020; Muhseen et al. 2020). The life cycle of a virus consists of four main steps, the first in which the virus identifies specific receptors and attaches them to the host cells. In the second stage, it fuses with the host cell membrane and inserts genetic material of them to the host cell. Next, the viral genetic material begins to produce structural and non-structured proteins using the host’s system, and finally the viral particles are assembled and separated from the host cell, already ready to attack the other cells (Chazal and Gerlier 2003; Choudhary et al. 2020). Thus, antiviral drugs targeting S-RBD-ACE2 interactions and M^pro^ inhibition, the early stage of infection, can provide a quick solution to combat viruses. For more rapid and effective findings, current led method is depending on the structural information of each target, rather than starting from a large, substrate molecule or numerous drug-sized molecules, starts with more limited smaller molecules, or fragments (Chazal and Gerlier 2003; Maveyraud and Mourey 2020). However, because the fragment or the compound is too small, they tend to easily bind to other proteins. these non-specific binding can lead to unpredictable toxicity. Thus, the small antiviral agents must be designed more precisely and accurately observed (Kelly et al. 2021). Therefore, considering above mentioned the advantages of cryo-EM and the specificity of this drug candidate screening, cryo-EM could be quickly and accurately determined molecular interactions in near native condition among several methods of binding ligands to proteins, and it can be powerful option as most effective starting point for in silico drug screening.

## Conclusions: future aspects of cryo-EM in drug discovery

Cryo-EM rapidly defined the high-resolution structure underlying SARS-CoV-2 drug development after the global pandemic outbreak, and many researchers are suggesting some notable candidates as a much-needed drug for SARS-CoV-2 by using this structural information (Panda et al. 2020; Wrapp et al. 2020). As has been much discussed now, various structural information by cryo-EM has provided unprecedented insights into the complex molecular mechanisms by dramatic technological achievement (Method of the year 2015 2016). Currently, by adopt of most structural biologists, drug design using structural information by cryo-EM is continuously increasing. A successful case is demonstrated on a paper by Wong et al., which identified an antimalarial mefloquine (MFQ) binding for 80S ribosome of plasmodium falciparum and used this information to structural improve an efficacy of MFQ (Wong et al. 2017). Unfortunately, of course, the representative advantage of cryo-EM is not applied that many samples can promptly observe high-resolution structural results. There are still key challenges to be resolved during structural analysis using cryo-EM. Nevertheless, this field is growing steadily, and cryo-EM is, by itself, a valuable technology in structural biology complementary to many traditional existing methods. If advances in software and hardware continue and further improve the efficiency of high throughput data analysis, such as automation of sample preparation, this will become a highly valuable technology development in structural biology and will greatly influence us to understand molecular events such as protein–ligand complexes, accelerating the drug discovery in near future.

## Data Availability

All data generated or analyzed during this study are included in this article and no datasets were generated or analyzed during the current study.

## Abbreviations

SARS-CoV-2: Severe acute respiratory syndrome coronavirus 2
ARDS: Acute respiratory distress syndrome
FBDD: Fragment-based drug discovery
Cryo-EM: Cryo-electron microscopy
SBDD: Structure-based drug design
NMR: Nuclear magnetic resonance
MD: Molecular dynamics
EM: Electron microscopy
GPCR: G-protein coupled receptor
TEM: Transmission electron microscope
S: Spike protein
RBD: Receptor binding domain
ACE2: Angiotensin-converting enzyme2
MERS: Middle East respiratory syndrome
SARS: Severe acute respiratory syndrome
HIV-1: Human immunodeficiency virus-1
Mpro: Main protease
RNA: Ribonucleic acid
MFQ: Mefloquine
